# Association of baseline peripheral sex hormone profiles and metabolic parameters with recurrence after oral isotretinoin treatment in patients with severe acne

**DOI:** 10.3389/fendo.2026.1912708

**Published:** 2026-09-14

**Authors:** Li-Fen Song, Yang Xiao, Hui Zhang

**Affiliations:** 1 Department of Dermatology, The Second Affiliated Hospital of Guangzhou University of Chinese Medicine (Guangdong Provincial Hospital of Traditional Chinese Medicine), Guangzhou, Guangdong, China; 2 Department of Urology, Dongguan Hospital of Traditional Chinese Medicine, Dongguan, Guangdong, China

**Keywords:** dehydroepiandrosterone sulfate, free androgen index, metabolic parameters, oral isotretinoin, recurrence, severe acne, sex hormones

## Abstract

**Background:**

Acne recurrence after oral isotretinoin remains common, but the clinical, treatment-related, hormonal, and metabolic factors associated with recurrence are not well defined.

**Methods:**

This retrospective cohort study included 351 patients (185 males and 166 females) with severe acne who received oral isotretinoin treatment between April 2021 and April 2025. Patients were classified into recurrence and non-recurrence groups according to follow-up outcomes after isotretinoin discontinuation. Baseline demographic characteristics, acne-related clinical features, isotretinoin treatment variables, peripheral sex hormone profiles, and metabolic parameters were collected. Univariate and multivariable logistic regression analyses were used to identify factors associated with recurrence. The combined prediction model was derived from the final multivariable logistic regression model and subsequently evaluated using receiver operating characteristic curve analysis, calibration assessment, and bootstrap internal validation.

**Results:**

During a median follow-up of 20.0 months, 91 patients developed recurrence, with an overall recurrence rate of 25.9%. Multivariate analysis showed that longer disease duration, lower cumulative isotretinoin dose, higher free androgen index, higher dehydroepiandrosterone sulfate, lower sex hormone-binding globulin, higher homeostatic model assessment of insulin resistance, higher triglyceride levels, and lower high-density lipoprotein cholesterol were independently associated with recurrence. Individual hormonal and metabolic indicators showed limited discriminatory ability. The combined prediction model demonstrated better performance, with an area under the curve of 0.824, sensitivity of 76.9%, and specificity of 76.2%. Bootstrap internal validation yielded a corrected area under the curve of 0.812.

**Conclusions:**

Individual hormonal and metabolic markers had limited discriminatory value, whereas a combined model integrating disease duration, cumulative isotretinoin dose, free androgen index, dehydroepiandrosterone sulfate, sex hormone-binding globulin, HOMA-IR, triglycerides, and high-density lipoprotein cholesterol showed better internal performance. Because cumulative dose becomes available during treatment, the model is best regarded as an exploratory dynamic risk-stratification tool; external prospective validation and a clearly defined management pathway are required before routine clinical use.

## 1 Introduction

Acne vulgaris is a chronic inflammatory disorder of the pilosebaceous unit and is characterized by comedones, inflammatory papules, pustules, nodules, cysts, and, in severe cases, permanent scarring (1). Although acne is traditionally regarded as a common dermatologic condition of adolescence, severe and persistent acne may reflect complex interactions among sebaceous gland activity, follicular hyperkeratinization, Cutibacterium acnes-related immune responses, skin barrier dysfunction, genetic susceptibility, and endocrine-metabolic regulation (2). The clinical burden of severe acne extends beyond visible skin lesions, as nodulocystic acne, acne conglobata, and acne with a high risk of scarring frequently require systemic therapy and long-term disease control strategies. Oral isotretinoin remains one of the most effective systemic treatments for severe acne because it targets several major pathogenic processes, including sebaceous gland activity, follicular keratinization, inflammation, and microbial changes within the pilosebaceous unit (3, 4). Current clinical recommendations support isotretinoin for severe, nodular, conglobate, treatment-resistant, or scar-prone acne (5). Nevertheless, relapse after treatment discontinuation remains clinically important, and differences in cumulative exposure, treatment duration, adherence, adverse event-related interruption, and baseline disease phenotype have been associated with post-treatment recurrence (6). Identifying factors associated with recurrence may therefore improve risk stratification and follow-up planning.

Endocrinological and metabolic abnormalities provide a plausible link between persistent acne activity and recurrence after apparent treatment response. Androgen signaling has a central role in sebocyte differentiation and sebum production, while insulin and insulin-like growth factor-1 pathways may enhance sebaceous lipogenesis, androgen receptor activity, and inflammatory signaling (7). Hormonal parameters such as total testosterone, free testosterone, free androgen index, dehydroepiandrosterone sulfate, and sex hormone-binding globulin may therefore provide clinically relevant information, particularly in patients with persistent or recurrent acne. In parallel, metabolic factors, including insulin resistance, dyslipidemia, and altered glucose metabolism, have been associated with acne severity and inflammatory activity (8). These observations support the hypothesis that baseline endocrine-metabolic status may influence the durability of remission after isotretinoin therapy.

Despite these advances, post-isotretinoin assessment in current practice remains centered on clinical severity, treatment response, treatment tolerance, and cumulative exposure, whereas laboratory-based prediction has not been established for routine use (9, 10). The present study therefore aimed to determine whether demographic and acne-related features, isotretinoin dose and duration, baseline peripheral sex hormone indices (including free androgen index, dehydroepiandrosterone sulfate, and sex hormone-binding globulin), and metabolic measures (including HOMA-IR and lipid parameters) were associated with recurrence, and to develop and internally validate a combined recurrence-risk model.

## 2 Methods

### 2.1 Study design and study population

This retrospective cohort study analyzed clinical and laboratory information that had been recorded prospectively during routine care for patients with severe acne treated with oral isotretinoin in the Department of Dermatology at our hospital between April 2021 and April 2025. During the study period, the institutional severe-acne pathway recommended baseline endocrine and metabolic assessment for all patients being considered for isotretinoin, including male patients; the present analysis was designed after care had been delivered. Eligible patients had complete baseline peripheral sex hormone and metabolic measurements before treatment and adequate follow-up after discontinuation. According to post-treatment outcomes, patients were classified into recurrence and non-recurrence groups.

Standardized follow-up data comprised dermatology clinic records and structured follow-up records after isotretinoin discontinuation. A minimum observation period of 12 months was predefined to provide a clinically meaningful opportunity to identify recurrence; adequate follow-up required a documented recurrence before 12 months or at least one assessment at or after month 12 with sufficient lesion and treatment information to assign recurrence status.

The study protocol was reviewed and approved by the Ethics Committee of our hospital. Secondary research use of anonymized clinical and laboratory data was documented for all included participants before data extraction. No additional study intervention or contact was performed for this retrospective analysis. All data were anonymized before analysis, and the study was conducted in accordance with the Declaration of Helsinki.

### 2.2 Diagnostic criteria for severe acne

Severe acne was diagnosed according to standard clinical dermatological criteria by qualified dermatologists. Disease severity was assessed using the six-category Investigator’s Global Assessment (IGA) scale used in the institutional record (0, clear; 1, almost clear; 2, mild; 3, moderate; 4, severe; and 5, very severe), rather than the FDA 0–4 IGA scale. Severe acne was therefore defined as an IGA score of 4 or 5. The treating dermatologist routinely assigned and recorded the baseline IGA score during the pre-isotretinoin visit; scores were not retrospectively reconstructed for this analysis. Clinical phenotypes included nodulocystic acne, acne conglobata, extensive inflammatory papules and pustules, deep nodules or cysts, and acne with an evident risk of permanent scarring.

### 2.3 Inclusion and exclusion criteria

Patients were included if they met all of the following criteria: (1) clinically diagnosed severe acne treated with oral isotretinoin at our institution; (2) baseline peripheral sex hormone and metabolic measurements obtained before the index course; (3) complete clinical, treatment, laboratory, and follow-up records; (4) completion of the prescribed course or a clinically evaluable treatment duration, defined as at least 4 months of therapy with documented cumulative dose and end-of-treatment response, including patients who discontinued prematurely; and (5) post-discontinuation follow-up meeting the adequate-follow-up definition above.

Patients were excluded for incomplete key data; systemic retinoid use during the 6 months preceding baseline laboratory assessment (with no retinoid exposure permitted between baseline testing and the index course); endocrine or metabolic disease that could materially alter hormone or metabolic measures; recent use of hormone-, glucose-, or lipid-modifying medication; major hepatic or renal dysfunction; or inadequate follow-up. Endocrine disorders were identified from established diagnoses and the baseline clinical and laboratory screen. Newly detected abnormalities prompted endocrinology or gynecology review and, when a relevant disorder was confirmed, exclusion. Polycystic ovary syndrome (PCOS) was assessed using the Rotterdam framework (two of oligo/anovulation, clinical or biochemical hyperandrogenism, and polycystic ovarian morphology after exclusion of alternative causes). All women with irregular cycles or amenorrhea underwent targeted clinical and biochemical evaluation; pelvic ultrasonography was performed when clinically indicated and was not mandatory in every female participant.

### 2.4 Oral isotretinoin treatment protocol

Oral isotretinoin was administered according to routine dermatological practice and individualized according to body weight, acne severity, clinical response, and treatment tolerance. The initial dose was generally 0.25-0.50 mg/kg/day, followed by adjustment according to response and adverse events. When tolerated, a cumulative exposure of approximately 120–150 mg/kg was considered the conventional target; however, treatment could end earlier after sustained clinical remission or because of intolerance. Topical retinoids and/or benzoyl peroxide were used selectively for persistent comedonal or inflammatory lesions during isotretinoin therapy, generally at reduced frequency and adjusted to cutaneous tolerance; their use was not protocol-mandated. Treatment data included initial and average daily dose, duration, cumulative dose, dose modification, concomitant therapy, interruption, premature discontinuation, end-of-treatment response, and adverse events.

### 2.5 Definition of recurrence and patient grouping

Acne recurrence was defined as reappearance of clinically significant inflammatory acne after isotretinoin discontinuation. Recurrence required at least one of the following: (1) recurrent moderate-to-severe inflammatory papules, pustules, nodules, or cysts; (2) a clear increase in severity relative to end-of-treatment status; or (3) initiation of further systemic anti-acne therapy, including systemic antibiotics, hormone-related therapy, or a second isotretinoin course. A record stating ‘recurrence’ or ‘relapse’ was accepted only when the same dermatologist note described lesions, worsening, or treatment that satisfied at least one of criteria 1-3; documentation alone without corroborating clinical information was not sufficient. Follow-up began at isotretinoin discontinuation and ended at first recurrence, last available assessment, or study end. All included patients had either documented recurrence or at least 12 months of evaluable follow-up.

### 2.6 Data collection

Patient data were obtained from the electronic medical record, laboratory information system, and standardized follow-up records. Two investigators independently used a predefined form, with discrepancies resolved against source records or by a third investigator. Variables included demographics, acne phenotype and severity, treatment characteristics, baseline hormonal and metabolic measures, follow-up duration, time to recurrence, and recurrence status. Post-discontinuation maintenance therapy initiated within 4 weeks after stopping isotretinoin was categorized as topical retinoid, benzoyl peroxide, topical antibiotic, or other topical therapy. Oral antibiotic exposure occurring before documented recurrence or censoring was also recorded. These variables were compared between groups and any maintenance therapy was included in the time-to-event sensitivity model.

Baseline peripheral sex hormone profiles included total testosterone, free testosterone or free androgen index, estradiol, luteinizing hormone, follicle-stimulating hormone, prolactin, progesterone, dehydroepiandrosterone sulfate, and sex hormone-binding globulin. Women with regular cycles were sampled during days 2–5 of the early follicular phase after an overnight fast. Women with irregular cycles or amenorrhea had cycle status, sampling date, and the targeted endocrine evaluation described above documented. Male participants were sampled in the morning after an overnight fast without cycle-related timing restrictions. Metabolic parameters included fasting glucose and insulin, HOMA-IR, total cholesterol, triglycerides, low- and high-density lipoprotein cholesterol, uric acid, and liver function indicators.

### 2.7 Laboratory measurements

All laboratory variables were measured before the initiation of oral isotretinoin therapy. Baseline laboratory assessment was defined as the most recent test performed within 1 month before treatment initiation. Peripheral venous blood samples were collected in the morning after an overnight fast to reduce the potential influence of food intake and circadian variation on metabolic parameters and selected hormone levels. Serum sex hormone levels were measured using chemiluminescence immunoassay or electrochemiluminescence immunoassay. Metabolic parameters, including fasting plasma glucose, lipid profiles, liver function indicators, and renal function indicators, were measured using an automated biochemical analyzer. Fasting insulin was measured using an immunoassay method. Insulin resistance was assessed using the homeostatic model assessment of insulin resistance (HOMA-IR), calculated as follows: HOMA-IR = fasting insulin (μIU/mL) × fasting plasma glucose (mmol/L)/22.5. All laboratory tests were performed by the Department of Laboratory Medicine at our hospital in accordance with standard operating procedures, and internal quality control measures were implemented throughout the testing process.

### 2.8 Statistical analysis

All analyses were performed using IBM SPSS Statistics, version 28.0 (IBM Corp., Armonk, NY, USA). Continuous variables were assessed for distribution and summarized as mean +/- standard deviation or median (interquartile range), as appropriate; categorical variables were reported as frequencies and percentages. Groups were compared using the independent-samples t test, Pearson chi-square test, or Fisher exact test, as appropriate. Univariable logistic regression identified candidate factors. Variables with P < 0.10, prespecified clinically relevant variables, and sex were considered for multivariable logistic regression; sex was retained as an adjustment covariate irrespective of its univariable P value.

To limit multicollinearity, correlated representations of the same construct were not entered simultaneously. Treatment duration was excluded when cumulative dose was included, and fasting insulin was excluded when HOMA-IR was included. Among total testosterone, free testosterone, free androgen index (FAI), and sex hormone-binding globulin (SHBG), candidate specifications were compared and the final model retained FAI and SHBG; total and free testosterone were not entered concurrently with FAI. Variance inflation factors were examined for all final-model terms. As sensitivity analyses, sex-stratified logistic models were fitted for FAI, DHEA-S, and SHBG with adjustment for disease duration and cumulative dose, and sex-by-marker interaction terms were evaluated in the full cohort.

Receiver operating characteristic analysis was used to assess individual indicators and the domain-specific and combined logistic models. Cumulative isotretinoin dose was additionally evaluated as a single predictor, with the optimal cutoff selected by the maximum Youden index. Pairwise AUC differences between the combined model and each domain-specific model were tested using DeLong’s method. The combined model was derived from the final multivariable equation; discrimination, calibration, Brier score, and 1000-resample bootstrap internal validation were evaluated.

To address bias from unequal follow-up duration, a multivariable Cox proportional hazards sensitivity analysis used time from isotretinoin discontinuation to first recurrence; patients without recurrence were censored at their last evaluable assessment. The model included the final logistic-regression predictors, sex, and post-treatment maintenance therapy. Hazard ratios (HRs) and 95% confidence intervals were reported. Decision curve analysis remained exploratory and defined a positive prediction as triggering closer dermatologic follow-up and consideration of guideline-concordant maintenance topical therapy, rather than automatic prolongation or retreatment with isotretinoin. All tests were two-sided, with P < 0.05 indicating statistical significance.

## 3 Results

### 3.1 Patient selection and follow-up outcomes

A total of 367 patients with severe acne who received oral isotretinoin treatment between April 2021 and April 2025 were initially screened for eligibility. Among them, 16 patients were excluded from the analysis for the following reasons: missing baseline sex hormone or metabolic parameters (n = 5), incomplete clinical or treatment records (n = 3), use of systemic retinoids within 6 months before the index course of isotretinoin (n = 2), presence of endocrine or metabolic disorders that could affect sex hormone or metabolic profiles (n = 3), and follow-up duration of less than 12 months or inability to determine recurrence status (n = 3). Finally, 351 patients were included in the final analysis. According to follow-up outcomes after discontinuation of oral isotretinoin, 91 patients were assigned to the recurrence group and 260 patients were assigned to the non-recurrence group. The overall recurrence rate was 25.9%. The median follow-up duration after isotretinoin discontinuation was 20.0 months, with an interquartile range of 15.0–28.0 months. Among patients who developed recurrence, the median time from isotretinoin discontinuation to first documented recurrence was 8.5 months, with an interquartile range of 5.0–13.0 months. The patient-selection process and reasons for exclusion are summarized in **Figure 1**.

**Figure 1:**
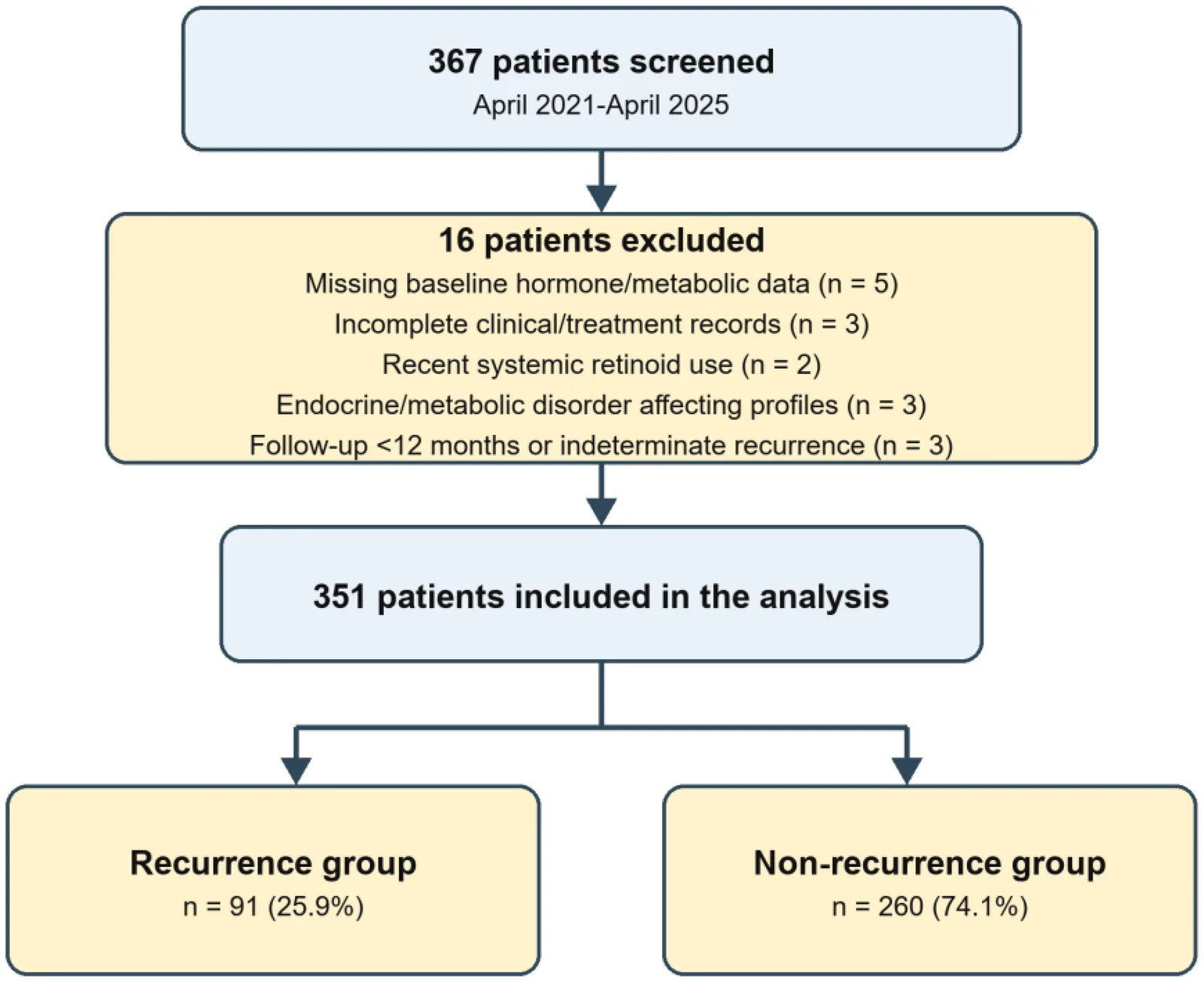
Patient-selection flow diagram. A total of 367 patients were screened, 16 were excluded for prespecified reasons, and 351 were included (91 with recurrence and 260 without recurrence).

### 3.2 Comparison of demographic and clinical characteristics between the recurrence and non-recurrence groups

The baseline demographic and clinical characteristics of patients in the recurrence and non-recurrence groups are shown in **Table 1**. There were no statistically significant differences between the two groups in age, sex distribution, body weight, height, body mass index, smoking status, alcohol consumption, menstrual status, previous acne treatment, or comorbidities (all P > 0.05). Compared with patients in the non-recurrence group, those in the recurrence group had a significantly longer disease duration (5.4 ± 2.6 vs. 4.1 ± 2.2 years; t = 4.62, P < 0.001) and a higher baseline Investigator’s Global Assessment score (4.42 ± 0.50 vs. 4.29 ± 0.46; t = 2.27, P = 0.024). The distribution of lesion type also differed significantly between the two groups (χ² = 12.13, P = 0.002), with higher proportions of nodulocystic acne and acne conglobata in the recurrence group. In addition, patients with recurrence were more likely to have facial and truncal involvement (60.4% vs. 48.5%; χ² = 3.87, P = 0.049), scar formation (62.6% vs. 45.4%; χ² = 8.03, P = 0.005), and a family history of acne (47.3% vs. 33.1%; χ² = 5.83, P = 0.016).

**Table 1:** Baseline demographic and clinical characteristics of patients in the recurrence and non-recurrence groups.

Variable	Recurrence group (n = 91)	Non-recurrence group (n = 260)	Statistic	P value
Age, years	22.8 ± 4.7	22.1 ± 4.5	t = 1.26	0.208
Male sex, n (%)	49 (53.8)	136 (52.3)	χ² = 0.06	0.800
Body weight, kg	61.8 ± 10.2	60.5 ± 9.8	t = 1.08	0.282
Height, cm	168.1 ± 8.4	167.4 ± 8.1	t = 0.70	0.483
Body mass index, kg/m²	21.8 ± 2.8	21.6 ± 2.7	t = 0.60	0.547
Disease duration, years	5.4 ± 2.6	4.1 ± 2.2	t = 4.62	<0.001
Baseline IGA score	4.42 ± 0.50	4.29 ± 0.46	t = 2.27	0.024
Lesion type, n (%)			χ² = 12.13	0.002
Severe inflammatory acne	24 (26.4)	119 (45.8)	—	—
Nodulocystic acne	52 (57.1)	119 (45.8)	—	—
Acne conglobata	15 (16.5)	22 (8.5)	—	—
Lesion distribution, n (%)			χ² = 3.87	0.049
Face only	36 (39.6)	134 (51.5)	—	—
Face and trunk	55 (60.4)	126 (48.5)	—	—
Scar formation, n (%)	57 (62.6)	118 (45.4)	χ² = 8.03	0.005
Family history of acne, n (%)	43 (47.3)	86 (33.1)	χ² = 5.83	0.016
Smoking history, n (%)	26 (28.6)	54 (20.8)	χ² = 2.33	0.127
Alcohol consumption, n (%)	18 (19.8)	44 (16.9)	χ² = 0.38	0.538
Menstrual status among female patients, n (%)	n = 42	n = 124	χ² = 1.10	0.578
Regular menstrual cycles	24 (57.1)	82 (66.1)	—	—
Irregular menstrual cycles	15 (35.7)	35 (28.2)	—	—
Amenorrhea	3 (7.1)	7 (5.6)	—	—
Previous acne treatment, n (%)			χ² = 3.67	0.300
No prior treatment	13 (14.3)	57 (21.9)	—	—
Topical treatment only	34 (37.4)	103 (39.6)	—	—
Systemic antibiotics	32 (35.2)	73 (28.1)	—	—
Other treatments	12 (13.2)	27 (10.4)	—	—
Comorbidities, n (%)	11 (12.1)	24 (9.2)	χ² = 0.61	0.434

### 3.3 Comparison of oral isotretinoin treatment characteristics between the two groups

The oral isotretinoin treatment characteristics of the two groups are presented in **Table 2**. No significant difference was observed between the recurrence and non-recurrence groups in the initial isotretinoin dose (0.33 ± 0.09 vs. 0.34 ± 0.08 mg/kg/day; t = -0.99, P = 0.321). However, the recurrence group had a lower average daily dose than the non-recurrence group (0.41 ± 0.12 vs. 0.45 ± 0.13 mg/kg/day; t = -2.58, P = 0.010). The treatment duration was significantly shorter in the recurrence group than in the non-recurrence group (7.8 ± 2.3 vs. 9.4 ± 2.5 months; t = -5.36, P < 0.001). Similarly, the cumulative isotretinoin dose was significantly lower in the recurrence group (103.6 ± 25.8 vs. 124.5 ± 28.6 mg/kg; t = -6.15, P < 0.001). Patients in the recurrence group had higher rates of temporary treatment interruption (24.2% vs. 13.5%; χ² = 5.69, P = 0.017) and premature discontinuation (17.6% vs. 8.1%; χ² = 6.46, P = 0.011). The use of concomitant acne therapies and the overall incidence of treatment-related adverse events were comparable between the two groups.

**Table 2:** Treatment characteristics of oral isotretinoin in the recurrence and non-recurrence groups.

Variable	Recurrence group (n = 91)	Non-recurrence group (n = 260)	Statistic	P value
Initial isotretinoin dose, mg/kg/day	0.33 ± 0.09	0.34 ± 0.08	t = -0.99	0.321
Average daily isotretinoin dose, mg/kg/day	0.41 ± 0.12	0.45 ± 0.13	t = -2.58	0.010
Treatment duration, months	7.8 ± 2.3	9.4 ± 2.5	t = -5.36	<0.001
Cumulative isotretinoin dose, mg/kg	103.6 ± 25.8	124.5 ± 28.6	t = -6.15	<0.001
Dose adjustment during treatment, n (%)	39 (42.9)	95 (36.5)	χ² = 1.14	0.286
Temporary treatment interruption, n (%)	22 (24.2)	35 (13.5)	χ² = 5.69	0.017
Premature discontinuation, n (%)	16 (17.6)	21 (8.1)	χ² = 6.46	0.011
Any concomitant acne therapy, n (%)	62 (68.1)	174 (66.9)	χ² = 0.04	0.833
Concomitant topical retinoids, n (%)	50 (54.9)	153 (58.8)	χ² = 0.42	0.517
Concomitant benzoyl peroxide, n (%)	42 (46.2)	136 (52.3)	χ² = 1.02	0.312
Concomitant topical antibiotics, n (%)	31 (34.1)	83 (31.9)	χ² = 0.14	0.707
Concomitant systemic antibiotics, n (%)	19 (20.9)	34 (13.1)	χ² = 3.20	0.074
Any treatment-related adverse event, n (%)	70 (76.9)	185 (71.2)	χ² = 1.13	0.288
Cheilitis, n (%)	63 (69.2)	174 (66.9)	χ² = 0.16	0.686
Xerosis, n (%)	47 (51.6)	123 (47.3)	χ² = 0.51	0.476
Abnormal liver function, n (%)	9 (9.9)	19 (7.3)	χ² = 0.61	0.434
Hypertriglyceridemia, n (%)	14 (15.4)	33 (12.7)	χ² = 0.42	0.516
Gastrointestinal symptoms, n (%)	7 (7.7)	15 (5.8)	χ² = 0.42	0.515
Musculoskeletal symptoms, n (%)	10 (11.0)	25 (9.6)	χ² = 0.14	0.707

### 3.4 Comparison of baseline peripheral sex hormone profiles between the recurrence and non-recurrence groups

Compared with patients in the non-recurrence group, patients in the recurrence group had significantly higher total testosterone levels (9.6 ± 7.5 vs. 7.8 ± 7.1 nmol/L; t = 2.00, P = 0.048), free testosterone levels (14.2 ± 10.4 vs. 11.5 ± 9.6 pg/mL; t = 2.17, P = 0.031), free androgen index (6.8 ± 5.2 vs. 5.2 ± 4.6; t = 2.60, P = 0.010), luteinizing hormone levels (6.8 ± 3.2 vs. 5.9 ± 2.9 IU/L; t = 2.36, P = 0.019), and dehydroepiandrosterone sulfate levels (7.3 ± 3.2 vs. 6.2 ± 2.9 μmol/L; t = 2.89, P = 0.004). In contrast, sex hormone-binding globulin levels were significantly lower in the recurrence group than in the non-recurrence group (39.5 ± 18.2 vs. 46.8 ± 19.6 nmol/L; t = -3.23, P = 0.002). No statistically significant differences were observed between the two groups in estradiol, follicle-stimulating hormone, prolactin, or progesterone levels. For female patients with regular menstrual cycles, sex hormone measurements were obtained during the early follicular phase, defined as days 2–5 of the menstrual cycle, to reduce the influence of physiological hormonal fluctuation (**Table 3**).

**Table 3:** Baseline peripheral sex hormone profiles in the recurrence and non-recurrence groups.

Variable	Recurrence group (n = 91)	Non-recurrence group (n = 260)	Statistic	P value
Total testosterone, nmol/L	9.6 ± 7.5	7.8 ± 7.1	t = 2.00	0.048
Free testosterone, pg/mL	14.2 ± 10.4	11.5 ± 9.6	t = 2.17	0.031
Free androgen index, %	6.8 ± 5.2	5.2 ± 4.6	t = 2.60	0.010
Estradiol, pmol/L	178.6 ± 96.2	169.4 ± 91.5	t = 0.80	0.428
Luteinizing hormone, IU/L	6.8 ± 3.2	5.9 ± 2.9	t = 2.36	0.019
Follicle-stimulating hormone, IU/L	5.9 ± 2.6	5.7 ± 2.5	t = 0.64	0.525
Prolactin, ng/mL	15.8 ± 6.8	14.6 ± 6.4	t = 1.47	0.143
Progesterone, nmol/L	1.82 ± 1.18	1.69 ± 1.10	t = 0.92	0.359
Dehydroepiandrosterone sulfate, μmol/L	7.3 ± 3.2	6.2 ± 2.9	t = 2.89	0.004
Sex hormone-binding globulin, nmol/L	39.5 ± 18.2	46.8 ± 19.6	t = -3.23	0.002

FAI, free androgen index; DHEA-S, dehydroepiandrosterone sulfate; SHBG, sex hormone-binding globulin.

### 3.5 Comparison of baseline metabolic parameters between the recurrence and non-recurrence groups

Compared with the non-recurrence group, the recurrence group had significantly higher fasting plasma glucose levels (5.05 ± 0.52 vs. 4.91 ± 0.48 mmol/L; t = 2.34, P = 0.020), fasting insulin levels (11.8 ± 4.7 vs. 9.6 ± 4.1 μIU/mL; t = 4.24, P < 0.001), and HOMA-IR values (2.67 ± 1.18 vs. 2.10 ± 0.98; t = 4.52, P < 0.001). Regarding lipid metabolism, patients in the recurrence group showed higher total cholesterol, triglyceride, and low-density lipoprotein cholesterol levels, whereas high-density lipoprotein cholesterol was significantly lower than that in the non-recurrence group. Uric acid was also slightly higher in the recurrence group (338.5 ± 82.4 vs. 319.2 ± 76.8 μmol/L; t = 2.02, P = 0.044). No statistically significant differences were observed between the two groups in baseline alanine aminotransferase, aspartate aminotransferase, gamma-glutamyl transferase, or creatinine levels, suggesting that baseline hepatic and renal function indicators were generally comparable between groups (**Table 4**).

**Table 4:** Baseline metabolic parameters in the recurrence and non-recurrence groups.

Variable	Recurrence group (n = 91)	Non-recurrence group (n = 260)	Statistic	P value
Fasting plasma glucose, mmol/L	5.05 ± 0.52	4.91 ± 0.48	t = 2.34	0.020
Fasting insulin, μIU/mL	11.8 ± 4.7	9.6 ± 4.1	t = 4.24	<0.001
HOMA-IR	2.67 ± 1.18	2.10 ± 0.98	t = 4.52	<0.001
Total cholesterol, mmol/L	4.72 ± 0.82	4.48 ± 0.78	t = 2.49	0.013
Triglycerides, mmol/L	1.51 ± 0.68	1.23 ± 0.56	t = 3.87	<0.001
Low-density lipoprotein cholesterol, mmol/L	2.78 ± 0.66	2.55 ± 0.61	t = 3.03	0.003
High-density lipoprotein cholesterol, mmol/L	1.17 ± 0.28	1.30 ± 0.31	t = -3.53	<0.001
Uric acid, μmol/L	338.5 ± 82.4	319.2 ± 76.8	t = 2.02	0.044
Alanine aminotransferase, U/L	22.8 ± 10.6	21.4 ± 9.8	t = 1.15	0.252
Aspartate aminotransferase, U/L	20.7 ± 7.8	20.1 ± 7.4	t = 0.66	0.512
Gamma-glutamyl transferase, U/L	23.5 ± 11.6	21.6 ± 10.8	t = 1.42	0.157
Creatinine, μmol/L	66.8 ± 13.5	65.1 ± 12.8	t = 1.07	0.283

### 3.6 Univariate logistic regression analysis of factors associated with acne recurrence

Univariate logistic regression analysis was performed to identify variables potentially associated with acne recurrence after oral isotretinoin treatment. As shown in **Table 5**, longer disease duration, higher baseline IGA score, nodulocystic or conglobate lesion type, scar formation, family history of acne, shorter treatment duration, lower cumulative isotretinoin dose, temporary treatment interruption, and premature discontinuation were associated with recurrence. In addition, several baseline hormonal and metabolic indicators, including free androgen index, DHEA-S, SHBG, fasting insulin, HOMA-IR, triglycerides, LDL-C, and HDL-C, were significantly associated with recurrence. Variables with P < 0.10 in univariate analysis, together with clinically relevant variables including age, sex, disease duration, baseline acne severity, treatment duration, and cumulative isotretinoin dose, were considered for subsequent multivariate logistic regression analysis.

**Table 5:** Univariate logistic regression analysis of candidate factors associated with acne recurrence after oral isotretinoin treatment.

Variable	OR	95% CI	P value
Age, per 1 year	1.03	0.98–1.09	0.208
Male sex	1.06	0.66–1.72	0.800
Disease duration, per 1 year	1.27	1.14–1.42	<0.001
Baseline IGA score, per 1 point	1.80	1.08–3.02	0.025
Nodulocystic/acne conglobata lesion type	2.36	1.39–3.99	0.001
Face and trunk involvement	1.62	1.00–2.64	0.050
Scar formation	2.02	1.24–3.29	0.005
Family history of acne	1.81	1.11–2.95	0.016
Smoking history	1.53	0.89–2.63	0.128
Treatment duration, per 1 month	0.76	0.69–0.85	<0.001
Cumulative isotretinoin dose, per 10 mg/kg	0.76	0.69–0.84	<0.001
Temporary treatment interruption	2.05	1.13–3.73	0.019
Premature discontinuation	2.43	1.21–4.89	0.013
Concomitant systemic antibiotics	1.75	0.94–3.26	0.076
Total testosterone, per 1 nmol/L	1.04	1.00–1.07	0.042
Free androgen index, per 1%	1.07	1.02–1.13	0.007
Luteinizing hormone, per 1 IU/L	1.11	1.02–1.20	0.015
DHEA-S, per 1 μmol/L	1.13	1.04–1.23	0.003
SHBG, per 10 nmol/L	0.82	0.72–0.93	0.002
Fasting insulin, per 1 μIU/mL	1.13	1.06–1.20	<0.001
HOMA-IR, per 1 unit	1.69	1.33–2.16	<0.001
Triglycerides, per 1 mmol/L	2.21	1.45–3.35	<0.001
LDL-C, per 1 mmol/L	1.81	1.22–2.68	0.003
HDL-C, per 0.1 mmol/L	0.87	0.80–0.94	<0.001

IGA, Investigator’s Global Assessment; OR, odds ratio; CI, confidence interval; DHEA-S, dehydroepiandrosterone sulfate; SHBG, sex hormone-binding globulin; HOMA-IR, homeostatic model assessment of insulin resistance; LDL-C, low-density lipoprotein cholesterol; HDL-C, high-density lipoprotein cholesterol.

### 3.7 Multivariate logistic regression analysis of independent risk factors for recurrence

Variables with P < 0.10 and clinically relevant covariates, including sex, were considered in the multivariable model. Treatment duration was not entered with cumulative dose, and fasting insulin was not entered with HOMA-IR. Total testosterone and free testosterone were not included concurrently with FAI; FAI and SHBG were retained because their variance inflation factors remained acceptable. After adjustment for sex and the other final-model covariates, longer disease duration, lower cumulative isotretinoin dose, higher FAI, higher dehydroepiandrosterone sulfate, lower SHBG, higher HOMA-IR, higher triglycerides, and lower high-density lipoprotein cholesterol remained independently associated with recurrence. Variance inflation factors ranged from 1.08 to 2.46, indicating no problematic multicollinearity (**Table 6**).

**Table 6:** Multivariate logistic regression analysis of independent factors associated with acne recurrence.

Variable	Adjusted OR	95% CI	Wald χ²	P value
Age, per 1 year	1.01	0.95–1.07	0.11	0.743
Male sex	1.12	0.62–2.03	0.14	0.708
Disease duration, per 1 year	1.18	1.05–1.34	7.08	0.008
Baseline IGA score, per 1 point	1.42	0.76–2.65	1.21	0.271
Nodulocystic/acne conglobata lesion type	1.71	0.95–3.08	3.20	0.074
Scar formation	1.62	0.92–2.86	2.78	0.095
Family history of acne	1.54	0.88–2.71	2.26	0.132
Cumulative isotretinoin dose, per 10 mg/kg	0.80	0.71–0.90	13.61	<0.001
Premature discontinuation	1.91	0.86–4.25	2.52	0.112
Free androgen index, per 1%	1.08	1.01–1.15	5.40	0.020
DHEA-S, per 1 μmol/L	1.10	1.00–1.21	3.84	0.050
SHBG, per 10 nmol/L	0.86	0.74–0.99	4.13	0.042
HOMA-IR, per 1 unit	1.45	1.09–1.92	6.62	0.010
Triglycerides, per 1 mmol/L	1.63	1.02–2.61	4.16	0.042
HDL-C, per 0.1 mmol/L	0.91	0.83–0.99	4.40	0.036

IGA, Investigator’s Global Assessment; OR, odds ratio; CI, confidence interval; DHEA-S, dehydroepiandrosterone sulfate; SHBG, sex hormone-binding globulin; HOMA-IR, homeostatic model assessment of insulin resistance; HDL-C, high-density lipoprotein cholesterol.

### 3.8 Predictive performance of individual hormonal and metabolic indicators for recurrence

ROC curve analysis was performed to evaluate the predictive performance of individual baseline hormonal and metabolic indicators for acne recurrence after oral isotretinoin treatment. The results are shown in **Table 7**. Among the evaluated indicators, HOMA-IR showed the highest predictive performance, with an AUC of 0.694 (95% CI: 0.634–0.754; Z = 6.34, P < 0.001), followed by fasting insulin (AUC = 0.681, 95% CI: 0.620–0.742; Z = 5.82, P < 0.001) and triglycerides (AUC = 0.662, 95% CI: 0.600–0.724; Z = 5.12, P < 0.001). Among hormonal indicators, free androgen index showed a moderate predictive value for recurrence (AUC = 0.646, 95% CI: 0.582–0.710; Z = 4.47, P < 0.001). DHEA-S, SHBG, HDL-C, LDL-C, and total testosterone also demonstrated statistically significant but relatively limited individual predictive performance. Overall, no single hormonal or metabolic indicator showed high discriminatory ability, suggesting that these variables may be more appropriate as components of a combined prediction model rather than as independent screening tools.

**Table 7:** ROC analysis of individual peripheral sex hormone and metabolic indicators for predicting acne recurrence.

Variable	AUC	95% CI	Z statistic	P value	Optimal cutoff	Sensitivity, %	Specificity, %
Total testosterone, nmol/L	0.612	0.546–0.678	3.33	<0.001	≥8.5	56.0	60.4
Free androgen index, %	0.646	0.582–0.710	4.47	<0.001	≥5.9	59.3	66.5
DHEA-S, μmol/L	0.632	0.568–0.696	4.04	<0.001	≥6.8	58.2	63.8
SHBG, nmol/L	0.637	0.573–0.700	4.23	<0.001	≤42.5	61.5	62.3
Fasting insulin, μIU/mL	0.681	0.620–0.742	5.82	<0.001	≥10.6	64.8	63.5
HOMA-IR	0.694	0.634–0.754	6.34	<0.001	≥2.35	65.9	66.9
Triglycerides, mmol/L	0.662	0.600–0.724	5.12	<0.001	≥1.36	62.6	62.7
HDL-C, mmol/L	0.641	0.578–0.704	4.39	<0.001	≤1.22	64.8	58.5
LDL-C, mmol/L	0.623	0.558–0.688	3.71	<0.001	≥2.66	59.3	60.0

AUC, area under the receiver operating characteristic curve; CI, confidence interval; DHEA-S, dehydroepiandrosterone sulfate; SHBG, sex hormone-binding globulin; HOMA-IR, homeostatic model assessment of insulin resistance; HDL-C, high-density lipoprotein cholesterol; LDL-C, low-density lipoprotein cholesterol.

### 3.9 Development and evaluation of the combined prediction model

Four logistic models were evaluated. The clinical-treatment model included disease duration, IGA score, lesion type, cumulative isotretinoin dose, and premature discontinuation; the hormonal model included FAI, DHEA-S, and SHBG; the metabolic model included HOMA-IR, triglycerides, and HDL-C; and the combined model included disease duration, cumulative isotretinoin dose, FAI, DHEA-S, SHBG, HOMA-IR, triglycerides, and HDL-C. The combined model was derived from the final multivariable logistic regression equation: logit(P) = 0.223 + 0.166 x disease duration - 0.223 x cumulative isotretinoin dose/10 + 0.077 x FAI + 0.095 x DHEA-S - 0.151 x SHBG/10 + 0.372 x HOMA-IR + 0.489 x triglycerides - 0.094 x HDL-C/0.1, where P is the predicted probability of recurrence. Because cumulative dose becomes known during treatment, the combined model represents dynamic end-of-treatment risk assessment rather than a purely pretreatment model.

The combined model showed better discrimination than the clinical-treatment, hormonal, or metabolic models (**Table 8**, **Figure 2**). Its AUC was 0.824 (95% CI, 0.776-0.872), with 76.9% sensitivity and 76.2% specificity at a predicted-probability cutoff of 0.265. The bootstrap-corrected AUC was 0.812; the Hosmer-Lemeshow chi-square was 6.21 (P = 0.624), and the Brier score was 0.142. Exploratory decision curve analysis suggested greater net benefit than treat-all and treat-none strategies across thresholds of approximately 10%-55% when a positive prediction was linked to closer follow-up and consideration of maintenance topical therapy. The analysis did not test whether such model-guided management improves outcomes.

**Table 8:** Predictive performance of the combined model for acne recurrence.

Model	Main included variables	AUC	95% CI	Z statistic	P value	Optimal cutoff	Sensitivity, %	Specificity, %	Youden index	Accuracy, %	Hosmer–Lemeshow χ²	Calibration P value	Brier score
Clinical-treatment model	Disease duration, IGA score, lesion type, cumulative isotretinoin dose, premature discontinuation	0.755	0.697–0.813	8.62	<0.001	0.248	68.1	72.3	0.404	71.2	7.84	0.449	0.168
Hormonal model	Free androgen index, DHEA-S, SHBG	0.719	0.660–0.778	7.28	<0.001	0.256	65.9	68.1	0.340	67.5	8.17	0.417	0.176
Metabolic model	HOMA-IR, triglycerides, HDL-C	0.748	0.690–0.807	8.28	<0.001	0.261	69.2	70.4	0.396	70.1	6.94	0.543	0.169
Combined prediction model	Disease duration, cumulative isotretinoin dose, free androgen index, DHEA-S, SHBG, HOMA-IR, triglycerides, HDL-C	0.824	0.776–0.872	13.23	<0.001	0.265	76.9	76.2	0.531	76.4	6.21	0.624	0.142
Bootstrap-corrected combined model	Internal validation with 1000 bootstrap resamples	0.812	0.764–0.861	12.56	<0.001	—	—	—	—	—	—	—	0.149

AUC, area under the receiver operating characteristic curve; CI, confidence interval; IGA, Investigator’s Global Assessment; DHEA-S, dehydroepiandrosterone sulfate; SHBG, sex hormone-binding globulin; HOMA-IR, homeostatic model assessment of insulin resistance; HDL-C, high-density lipoprotein cholesterol.

**Figure 2:**
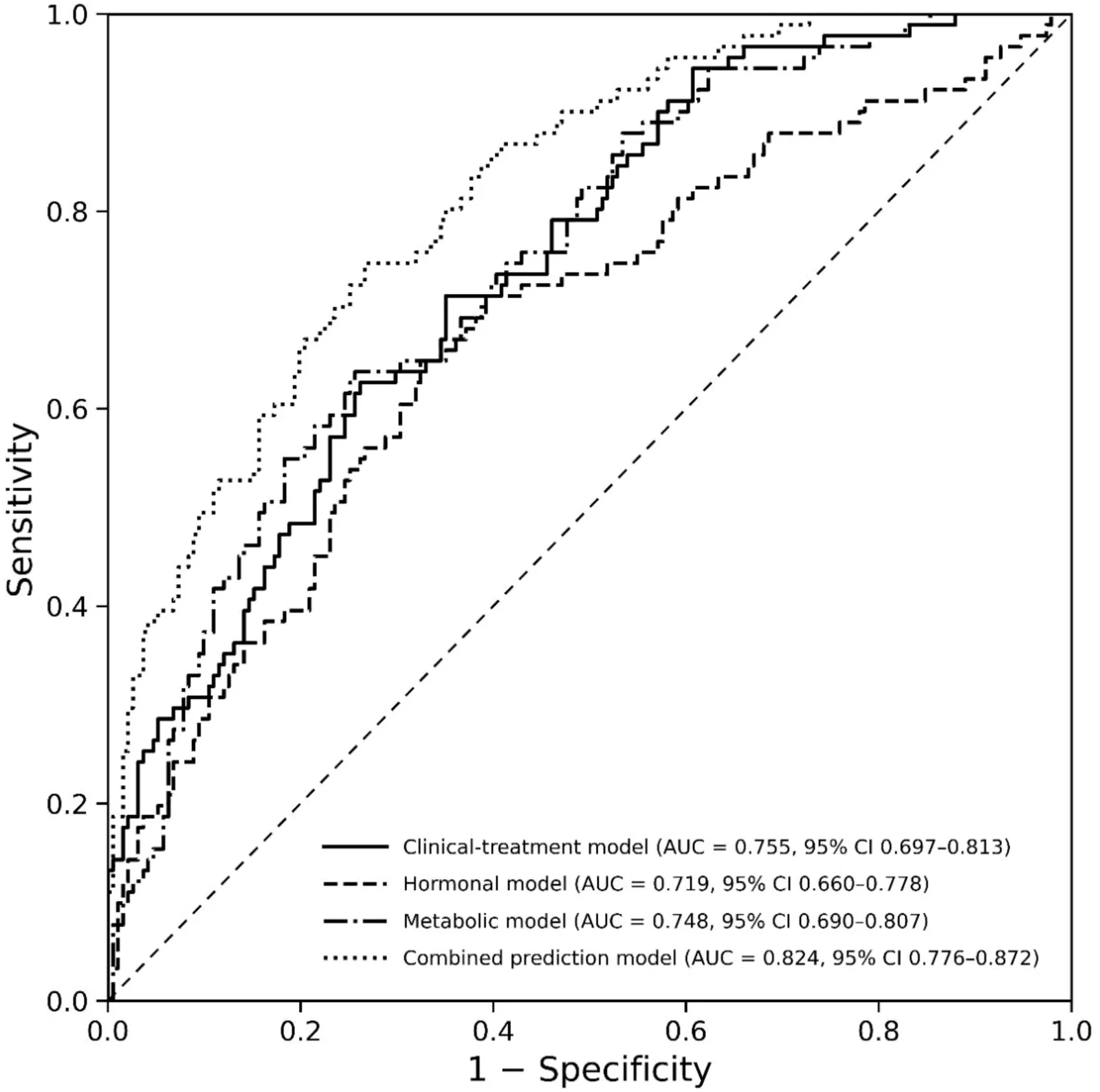
Predictive performance of clinical-treatment, hormonal, metabolic, and combined models for acne recurrence after oral isotretinoin treatment. Clinical-treatment: disease duration, IGA score, lesion type, cumulative isotretinoin dose, and premature discontinuation; hormonal: FAI, DHEA-S, and SHBG; metabolic: HOMA-IR, triglycerides, and HDL-C; combined: disease duration, cumulative isotretinoin dose, FAI, DHEA-S, SHBG, HOMA-IR, triglycerides, and HDL-C. The combined model had the highest AUC (0.824; 95% CI, 0.776-0.872).

### 3.10 Additional sensitivity analyses

Post-discontinuation treatment patterns were similar between groups (**Supplementary Table 1**). Any maintenance therapy was recorded in 61 of 91 patients with recurrence (67.0%) and 177 of 260 patients without recurrence (68.1%; P = 0.854). No significant between-group differences were observed for topical retinoids, benzoyl peroxide, topical antibiotics, or oral antibiotics used before recurrence or censoring. Among the 60 women with irregular cycles or amenorrhea, all underwent targeted clinical and biochemical assessment; pelvic ultrasonography was available for 48 (80.0%). None fulfilled two Rotterdam features after exclusion of alternative causes.

The Cox sensitivity analysis yielded results broadly consistent with the primary logistic model (**Supplementary Table 2**). Longer disease duration (HR, 1.11 per year; 95% CI, 1.02-1.21; P = 0.014), lower cumulative isotretinoin dose (HR, 0.81 per 10 mg/kg; 95% CI, 0.74-0.88; P < 0.001), higher FAI (HR, 1.07 per 1%; 95% CI, 1.03-1.12; P < 0.001), lower SHBG (HR, 0.89 per 10 nmol/L; 95% CI, 0.79-1.00; P = 0.042), higher HOMA-IR (HR, 1.41; 95% CI, 1.18-1.68; P < 0.001), higher triglycerides (HR, 1.55; 95% CI, 1.16-2.06; P = 0.003), and lower HDL-C (HR, 0.87 per 0.1 mmol/L; 95% CI, 0.81-0.94; P < 0.001) were associated with shorter recurrence-free time. DHEA-S retained the same direction but was not significant (HR, 1.02; 95% CI, 0.94-1.10; P = 0.616). Maintenance therapy was not associated with recurrence-free time (HR, 0.97; 95% CI, 0.61-1.55; P = 0.891).

Kaplan-Meier curves stratified by the descriptive cumulative-dose cutoff are provided in **Supplementary Figure 1** (log-rank P < 0.001); the full Cox estimates are shown in **Supplementary Figure 2**.

Sex-stratified sensitivity analyses showed similar effect directions (**Supplementary Table 3**, **Supplementary Figure 4**). FAI was associated with recurrence in both males (OR, 1.14; 95% CI, 1.04-1.25; P = 0.004) and females (OR, 1.18; 95% CI, 1.06-1.31; P = 0.003). No significant sex-by-marker interaction was observed for FAI (P for interaction = 0.673), DHEA-S (P = 0.745), or SHBG (P = 0.412), although some stratum-specific confidence intervals crossed unity because of reduced precision.

Cumulative isotretinoin dose alone showed modest discrimination (AUC, 0.698; 95% CI, 0.632-0.764), with a data-derived cutoff of ≤112.3 mg/kg yielding 67.0% sensitivity and 66.9% specificity (**Supplementary Table 4**, **Supplementary Figure 3**). DeLong tests confirmed that the combined model had a higher AUC than the clinical-treatment model (difference, 0.069; P = 0.014), hormonal model (difference, 0.105; P < 0.001), and metabolic model (difference, 0.076; P = 0.003; **Supplementary Table 5**). The cumulative-dose cutoff was considered descriptive and was not interpreted as a universal treatment target.

## 4 Discussion

The rationale for this study was that recurrence after isotretinoin is unlikely to be explained by treatment exposure alone. We therefore examined whether baseline clinical phenotype, endocrine-metabolic status, and isotretinoin exposure jointly characterize recurrence risk and whether their integration improves discrimination over isolated biomarkers. In this cohort, single hormonal and metabolic markers had limited predictive value, whereas the combined model showed better internally validated discrimination. These findings support a multifactorial and dynamic interpretation of recurrence risk, while not establishing a model-guided intervention.

Cumulative isotretinoin exposure requires particular consideration. The recurrence group received a mean cumulative dose of 103.6 +/- 25.8 mg/kg compared with 124.5 +/- 28.6 mg/kg in the non-recurrence group, and lower cumulative dose remained independently associated with recurrence. In supplemental ROC analysis, cumulative dose alone showed modest discrimination (AUC, 0.698), and the data-derived threshold of <=112.3 mg/kg had only 67.0% sensitivity and 66.9% specificity. These data are directionally consistent with large cohort evidence and the conventional cumulative target of approximately 120–150 mg/kg (11, 12), but the moderate discrimination and between-patient variability argue against interpreting the derived cutoff as a universal target. Cumulative exposure should remain individualized according to response, tolerability, adverse events, and adherence (13).

Longer recorded disease duration was associated with recurrence, but it should not be assumed to represent an intrinsically persistent biological phenotype. In a retrospective referral cohort, recorded duration may reflect age at onset, referral patterns, prior treatment choices, healthcare access, recall accuracy, and the interval between onset and isotretinoin initiation. Conversely, early-onset aggressive acne treated soon after onset may have a short recorded duration despite severe biology. Because age at onset and detailed access-to-care variables were not modeled, disease duration should be interpreted as a pragmatic clinical marker rather than a direct mechanistic measure.

The androgen-related findings provide a distinct biological signal. Higher FAI and DHEA-S and lower SHBG remained associated with recurrence after adjustment for sex, clinical factors, and treatment exposure. FAI and SHBG may reflect circulating bioavailable androgens more closely than total testosterone alone, while adrenal DHEA-S may contribute to sebaceous stimulation. Sex-stratified sensitivity analyses showed consistent effect directions and no significant sex-by-marker interactions, although reduced precision caused some stratum-specific estimates to cross unity. Insulin resistance may further increase androgen bioavailability by reducing hepatic SHBG production. Because physiological hormone distributions differ by sex and menstrual status, these associations should not be interpreted using a universal diagnostic threshold; sex-specific reference ranges and external validation remain necessary.

The relationship between insulin resistance and recurrence is another important observation. HOMA-IR was the strongest individual metabolic predictor in ROC analysis, although its single-marker discrimination remained limited. In multivariate analysis, higher HOMA-IR remained independently associated with recurrence, indicating that insulin resistance may contribute to post-isotretinoin relapse beyond baseline acne severity and cumulative dose. Recent studies have reported higher insulin levels and HOMA-IR values in patients with acne and have proposed insulin resistance as a contributing factor in acne development and severity (14, 15). Mechanistically, hyperinsulinemia may increase insulin-like growth factor-1 signaling, promote sebocyte lipogenesis, enhance androgen synthesis, and reduce hepatic SHBG production, thereby increasing bioavailable androgens. This interaction may explain why both HOMA-IR and androgen-related indices remained significant in the present model (16, 17). The findings suggest that metabolic status should not be considered separate from hormonal regulation in severe acne, but rather as part of a connected endocrine-metabolic network. Dyslipidemia also appeared to be associated with recurrence. Higher triglyceride levels and lower HDL-C levels remained independently associated with recurrence after multivariable adjustment. This finding is relevant because isotretinoin itself can influence lipid metabolism during treatment, but in this study the metabolic variables were assessed at baseline before treatment initiation. Therefore, the observed association more likely reflects pre-existing metabolic status rather than treatment-induced lipid changes. Recent reports have linked acne with metabolic syndrome, insulin resistance, and lipid abnormalities, including higher triglyceride levels and lower HDL-C levels in more severe acne phenotypes (18, 19). These metabolic features may contribute to chronic low-grade inflammation, altered sebaceous lipid composition, and sustained follicular inflammation. Nevertheless, lipid markers should not be interpreted as causal determinants of recurrence based on the present retrospective data.

The combined model achieved an AUC of 0.824 and maintained acceptable discrimination after bootstrap correction, whereas isolated markers generally had AUCs below 0.70. DeLong comparisons supported statistically higher discrimination for the combined model than for the clinical-treatment, hormonal, or metabolic models. Its clinical translation nevertheless has two important constraints. First, the model requires multiple laboratory variables and would need simplification, external validation, and presentation as a user-friendly nomogram or online calculator. Second, cumulative isotretinoin dose is not available at baseline; the model should therefore be viewed as a dynamic tool updated near treatment completion, not as a purely pretreatment score. A future tool could separate a baseline model from an end-of-treatment update and prospectively test whether risk-guided follow-up or maintenance therapy improves patient outcomes.

Clinically, a high predicted risk at treatment completion could reasonably trigger closer dermatologic follow-up, adherence counseling, and consideration of guideline-concordant maintenance topical therapy; it should not automatically trigger prolonged isotretinoin, retreatment, hormonal therapy, or metabolic treatment (20–22). Decision curve analysis was included only to explore the net benefit of this explicitly defined surveillance/maintenance strategy. Because no model-guided intervention was delivered or compared in this cohort, the DCA remains hypothesis-generating rather than evidence of clinical effectiveness.

Several limitations merit emphasis. The retrospective single-center design and complete-case requirement may introduce selection bias. Although the Cox sensitivity analysis accounted for censoring, follow-up intensity and healthcare-seeking behavior could still affect recurrence detection. Post-discontinuation therapies were observational rather than standardized; the absence of significant group differences does not exclude residual confounding. Pelvic ultrasonography was unavailable for 12 of 60 women with irregular cycles or amenorrhea, so subclinical PCOS could have been missed despite targeted evaluation. Hormone measures were baseline-only, and sex-stratified estimates had limited precision. Age at acne onset was not modeled separately from disease duration. Finally, the prediction model was internally validated only, the cumulative-dose threshold was data-derived, and the management action linked to DCA was not tested. Prospective multicenter studies with standardized post-treatment therapy and external validation are required.

## 5 Conclusion

In conclusion, longer disease duration, lower cumulative isotretinoin dose, higher FAI and DHEA-S, lower SHBG, higher HOMA-IR and triglycerides, and lower HDL-C were independently associated with recurrence after oral isotretinoin in this cohort. Individual biomarkers had limited discriminatory value, whereas the combined model showed better discrimination and calibration. Because the model includes cumulative dose, it is best considered an exploratory dynamic end-of-treatment risk tool. External prospective validation and evaluation of a prespecified model-guided follow-up or maintenance strategy are required before routine use.

## Data Availability

The raw data supporting the conclusions of this article will be made available by the authors, without undue reservation.
