# Correction: Association of baseline peripheral sex hormone profiles and metabolic parameters with recurrence after oral isotretinoin treatment in patients with severe acne

**DOI:** 10.3389/fendo.2026.2005509

**Published:** 2026-10-16

**Authors:** Li-Fen Song, Yang Xiao, Hui Zhang

**Affiliations:** 1 Department of Dermatology, The Second Affiliated Hospital of Guangzhou University of Chinese Medicine (Guangdong Provincial Hospital of Traditional Chinese Medicine), Guangzhou, Guangdong, China; 2 Department of Urology, Dongguan Hospital of Traditional Chinese Medicine, Dongguan, Guangdong, China

**Keywords:** dehydroepiandrosterone sulfate, free androgen index, metabolic parameters, oral isotretinoin, recurrence, severe acne, sex hormones

The **Ethics statement** was erroneously given as “The studies involving humans were approved by the Ethics Committee of Women’s Hospital of Nanjing Medical University(Nanjing Women and Children’s Healthcare Hospital). The studies were conducted in accordance with the local legislation and institutional requirements. The participants provided their written informed consent to participate in this study”. The correct **Ethics statement** is “The studies involving humans were approved by the Ethics Committee of The Guangdong Provincial Hospital of Traditional Chinese Medicine. The studies were conducted in accordance with the local legislation and institutional requirements. The participants provided their written informed consent to participate in this study”.

The original version of this article has been updated.

