# Supplementary material for: Association of baseline peripheral sex hormone profiles and metabolic parameters with recurrence after oral isotretinoin treatment in patients with severe acne
Source: Front Endocrinol (Lausanne). 2026 Sep 14;17:1912708. doi: 10.3389/fendo.2026.1912708 (PMC13616687; doi:10.3389/fendo.2026.1912708)
Supplement: Supplementary file 1 [file SupplementaryFile1.docx]

**Supplementary Material**

**Additional sensitivity analyses for acne recurrence after oral isotretinoin treatment**

**Supplementary Methods**

Time-to-event sensitivity analysis. Time from isotretinoin discontinuation to the first documented recurrence was analyzed using a multivariable Cox proportional hazards model. Patients without recurrence were censored at their last evaluable assessment. The model included the final predictors from the primary logistic model, sex, and any post-discontinuation maintenance therapy. Kaplan-Meier curves were additionally displayed after stratification by the descriptive cumulative-dose cutoff, with groups compared using a two-sided log-rank test.

Supplementary prediction analyses. Cumulative isotretinoin dose was evaluated as a single predictor using ROC analysis. The maximum Youden index defined the descriptive cutoff, and the AUC was reported with a 95% confidence interval. Pairwise AUC differences between the combined and domain-specific models were tested using DeLong's method.

Sex-stratified analyses. Exploratory sex-stratified logistic models were fitted for the free androgen index, DHEA-S, and SHBG, with adjustment for disease duration and cumulative isotretinoin dose. Sex-by-marker interaction terms were tested in the full cohort. All tests were two-sided.

**Supplementary Table S1. Post-discontinuation maintenance therapy in the recurrence and non-recurrence groups**

| **Therapy** | **Recurrence group (n = 91), n (%)** | **Non-recurrence group (n = 260), n (%)** | **P value** |
| --- | --- | --- | --- |
| Any maintenance therapy | 61 (67.0) | 177 (68.1) | 0.854 |
| Topical retinoid | 47 (51.6) | 145 (55.8) | 0.497 |
| Benzoyl peroxide | 34 (37.4) | 110 (42.3) | 0.409 |
| Topical antibiotic | 17 (18.7) | 44 (16.9) | 0.703 |
| Oral antibiotic before recurrence/censoring | 5 (5.5) | 11 (4.2) | 0.619 |

Treatments were recorded from isotretinoin discontinuation to recurrence or censoring, as applicable. P values compare proportions between groups.

**Supplementary Table S2. Multivariable Cox proportional hazards sensitivity analysis of time to acne recurrence**

| **Variable** | **Adjusted HR** | **95% CI** | **P value** |
| --- | --- | --- | --- |
| Disease duration, per 1 year | 1.11 | 1.02-1.21 | 0.014 |
| Cumulative isotretinoin dose, per 10 mg/kg | 0.81 | 0.74-0.88 | <0.001 |
| Free androgen index, per 1% | 1.07 | 1.03-1.12 | <0.001 |
| DHEA-S, per 1 micromol/L | 1.02 | 0.94-1.10 | 0.616 |
| SHBG, per 10 nmol/L | 0.89 | 0.79-1.00 | 0.042 |
| HOMA-IR, per 1 unit | 1.41 | 1.18-1.68 | <0.001 |
| Triglycerides, per 1 mmol/L | 1.55 | 1.16-2.06 | 0.003 |
| HDL-C, per 0.1 mmol/L | 0.87 | 0.81-0.94 | <0.001 |
| Male sex | 0.81 | 0.53-1.24 | 0.335 |
| Any post-treatment maintenance therapy | 0.97 | 0.61-1.55 | 0.891 |

HR, hazard ratio; CI, confidence interval; DHEA-S, dehydroepiandrosterone sulfate; SHBG, sex hormone-binding globulin; HOMA-IR, homeostatic model assessment of insulin resistance; HDL-C, high-density lipoprotein cholesterol.

**Supplementary Table S3. Sex-stratified sensitivity analyses of androgen-related predictors**

| **Variable** | **Sex** | **Adjusted OR** | **95% CI** | **P value** | **P for interaction** |
| --- | --- | --- | --- | --- | --- |
| Free androgen index, per 1% | Male | 1.14 | 1.04-1.25 | 0.004 | 0.673 |
| Free androgen index, per 1% | Female | 1.18 | 1.06-1.31 | 0.003 | 0.673 |
| DHEA-S, per 1 micromol/L | Male | 1.13 | 1.00-1.29 | 0.047 | 0.745 |
| DHEA-S, per 1 micromol/L | Female | 1.10 | 0.96-1.26 | 0.173 | 0.745 |
| SHBG, per 10 nmol/L | Male | 0.89 | 0.73-1.08 | 0.237 | 0.412 |
| SHBG, per 10 nmol/L | Female | 0.78 | 0.63-0.97 | 0.026 | 0.412 |

Models were adjusted for disease duration and cumulative isotretinoin dose. OR, odds ratio; CI, confidence interval; DHEA-S, dehydroepiandrosterone sulfate; SHBG, sex hormone-binding globulin.

**Supplementary Table S4. ROC analysis of cumulative isotretinoin dose for predicting acne recurrence**

| **Predictor** | **AUC** | **95% CI** | **Optimal cutoff** | **Sensitivity, %** | **Specificity, %** | **Youden index** |
| --- | --- | --- | --- | --- | --- | --- |
| Cumulative isotretinoin dose, mg/kg | 0.698 | 0.632-0.764 | <=112.3 mg/kg | 67.0 | 66.9 | 0.340 |

AUC, area under the receiver operating characteristic curve; CI, confidence interval. The data-derived cutoff is descriptive and should not be interpreted as a universal dosing target.

**Supplementary Table S5. DeLong comparisons of the combined model with domain-specific prediction models**

| **Comparison** | **Combined AUC** | **Comparator AUC** | **AUC difference** | **Z statistic** | **P value** |
| --- | --- | --- | --- | --- | --- |
| Combined vs Clinical-treatment | 0.824 | 0.755 | 0.069 | 2.45 | 0.014 |
| Combined vs Hormonal | 0.824 | 0.719 | 0.105 | 4.02 | <0.001 |
| Combined vs Metabolic | 0.824 | 0.748 | 0.076 | 2.95 | 0.003 |

AUC, area under the receiver operating characteristic curve. Positive differences favor the combined model.

**Supplementary Figure S1. Cumulative probability of acne recurrence stratified by cumulative isotretinoin dose**

Kaplan-Meier curves depict cumulative recurrence after isotretinoin discontinuation for patients receiving ≤112.3 mg/kg or >112.3 mg/kg. Censoring marks are shown, and the risk table reports the number remaining under observation. Groups were compared using a two-sided log-rank test (n = 351; 91 recurrence events). The cutoff was derived using the maximum Youden index and should be interpreted descriptively.

**Supplementary Figure S2. Multivariable Cox sensitivity analysis of time to acne recurrence**

Points show adjusted hazard ratios and horizontal lines show 95% confidence intervals. Filled points indicate P < 0.05; open points indicate P ≥ 0.05. The vertical dashed line denotes an HR of 1.0. The model included 351 patients and 91 recurrence events.

**Supplementary Figure S3. Discriminatory performance of cumulative isotretinoin dose**

The ROC curve evaluates cumulative isotretinoin dose as a single predictor of recurrence (n = 351; 91 recurrences). The orange point marks the maximum-Youden cutoff of ≤112.3 mg/kg, corresponding to 67.0% sensitivity and 66.9% specificity. The cutoff is descriptive and is not proposed as a universal dosing target.

**Supplementary Figure S4. Sex-stratified associations of androgen-related predictors with acne recurrence**

Points show adjusted odds ratios and horizontal lines show 95% confidence intervals for males (blue circles; n = 185) and females (orange squares; n = 166). Models were adjusted for disease duration and cumulative isotretinoin dose. Interaction P values test whether the marker association differed by sex.
